# Low Back Pain With Persistent Radiculopathy; the Clinical Role of Genetic Variants in the Genes SOX5, CCDC26/GSDMC and DCC

**DOI:** 10.3389/fgene.2021.757632

**Published:** 2022-01-24

**Authors:** Marie Udnesseter Lie, Linda Margareth Pedersen, Ingrid Heuch, Bendik Winsvold, Johannes Gjerstad, Eivind Hasvik, Øystein Petter Nygaard, Margreth Grotle, Dagfinn Matre, John-Anker Zwart, Kristian Bernhard Nilsen

**Affiliations:** ^1^ Research and Communication Unit for Musculoskeletal Health (FORMI), Division of Clinical Neuroscience, Oslo University Hospital, Oslo, Norway; ^2^ Faculty of Medicine, University of Oslo, Oslo, Norway; ^3^ Department of Research, Innovation and Education, Division of Clinical Neuroscience, Oslo University Hospital, Oslo, Norway; ^4^ Department of Physiotherapy, Oslo Metropolitan University, Oslo, Norway; ^5^ Department of Neurology, Oslo University Hospital, Oslo, Norway; ^6^ Department of Work Psychology and Physiology, National Institute of Occupational Health, Oslo, Norway; ^7^ Department of Bioscience, University of Oslo, Oslo, Norway; ^8^ Department of Physical Medicine and Rehabilitation, Østfold Hospital Trust, Grålum, Norway; ^9^ Department of Neurosurgery, St Olavs University Hospital, Trondheim, Norway; ^10^ Department of Neuroscience, Norwegian University of Science and Technology (NTNU), Trondheim, Norway; ^11^ National Advisory Unit on Spinal Surgery, St Olavs Hospital, Trondheim, Norway

**Keywords:** low back pain, lumbar radiculopathy, genetic susceptibility, biomarkers, candidate gene study (CGS)

## Abstract

In a recently published genome-wide association study (GWAS) chronic back pain was associated with three loci; *SOX5, CCDC26/GSDMC* and *DCC*. This GWAS was based on a heterogeneous sample of back pain disorders, and it is unknown whether these loci are of clinical relevance for low back pain (LBP) with persistent radiculopathy. Thus, we examine if LBP with radiculopathy 12 months after an acute episode of LBP with radiculopathy is associated with the selected single nucleotide polymorphisms (SNPs); *SOX5* rs34616559, *CCDC26/GSDMC* rs7833174 and *DCC* rs4384683. In this prospective cohort study, subjects admitted to a secondary health care institution due to an acute episode of LBP with radiculopathy, reported back pain, leg pain, and Oswestry Disability Index (ODI), were genotyped and followed up at 12 months (*n* = 338). Kruskal-Wallis H test showed no association between the SNPs and back pain, leg pain or ODI. In conclusion, LBP with radiculopathy 12 months after an acute episode of LBP with radiculopathy, is not associated with the selected SNPs; *SOX5* rs34616559, *CCDC26/GSDMC* rs7833174 and *DCC* rs4384683. This absent or weak association suggests that the SNPs previously associated with chronic back pain are not useful as prognostic biomarkers for LBP with persistent radiculopathy.

## Introduction

Low back pain (LBP) is the leading cause of years lived with disability in Western countries (Global Burden of Disease Study, 2020) and represents a large economic burden (Maniadakis and Gray, 2000; Breivik et al., 2013; Olafsson et al., 2018). Although the majority of patients with LBP recover within a few weeks, a significant number of patients develop chronic LBP (Hayden et al., 2010). One common condition of LBP is radiculopathy, in which the patient experiences pain and/or paresthesia in the distribution of the lumbar spinal nerve due to a nerve root compression (Stafford et al., 2007). Radiculopathy is more persistent, severe, has a less favorable outcome and consumes more health resources than LBP (Konstantinou and Dunn, 2008), and may therefore account for a considerable part of the socioeconomic burden of LBP. Thus, to reduce the socioeconomic burden of LBP, better treatment, monitoring and prevention of LBP with radiculopathy are needed. Genetic susceptibility is assumed to play an important role for LBP with radiculopathy (Williams et al., 2013; Bjorland et al., 2016; Lemmelä et al., 2016; Bjornsdottir et al., 2017). The use of such genetic information in personalized medicine holds great promise to improve health care. A genome-wide association study (GWAS) is a suitable approach to identify new associations between diseases and single nucleotide polymorphisms (SNPs) (Tam et al., 2019). However, genetic association studies with a candidate gene approach using smaller, but better described, patient samples with a detailed phenotyping is often necessary to evaluate the clinical value of findings from GWASs, such as whether SNPs can be used as prognostic biomarkers on an individual level. In the first reported GWAS meta-analysis of chronic back pain with 158.000 individuals three loci were identified in or near the genes SOX5, CCDC26/GSDMC and DCC (Suri et al., 2018). However, this GWAS comprised a heterogeneous sample of chronic back pain disorders. It is unknown whether these loci are of clinical relevance for LBP with persistent radiculopathy. Thus, we aimed to examine if LBP with radiculopathy 12 months after an acute episode of radiculopathy is associated with the selected SNPs; *SOX5* rs34616559, *CCDC26/GSDMC* rs7833174 and *DCC* rs4384683.

## Materials and Methods

### Design

The present study used data from a prospective cohort study of subjects which required admission to a secondary health care institution due to an acute episode of LBP with radiculopathy. Questionnaires, clinical and radiological examination, and blood samples were obtained at admission. Questionnaires were obtained 12 months after admission, either by post or at a follow-up assessment. If the 12-months questionnaire was not returned within 2 weeks, subjects were reminded by mail and/or phone. The institutions’ health care personnel assisted in inclusion, data collection and follow-up of patients, but the subjects were treated independently of the study with lumbar surgery and/or conservative treatment. The surgical approach and conservative treatment were individualized to the patient in agreement with neurology specialists following clinical guidelines.

A written informed consent was obtained prior to participation. The study was approved by the regional committee for medical and health research ethics (project number: 2013/1060) and conducted in accordance with the Declaration of Helsinki.

### Subjects

Subjects were recruited from three hospitals in Norway; 1) the Neurological Department at Oslo University Hospital between January 2013 and June 2018 (*n* = 301), 2) a secondary health care back clinic at the Østfold Hospital Trust between January 2014 and May 2016 (*n* = 86), and 3) Department of Neurosurgery at St. Olavs hospital, Trondheim between January 2015 and May 2016 (*n* = 50). Inclusion criteria were age 18 years or older and the presence of acute radiculopathy defined as acute LBP with radiating pain with dermatomal distribution corresponding to radiological findings of lumbar nerve root compression confirmed by either MRI or CT. Exclusion criteria were non-Caucasian heritage (mother and father), unable to understand spoken and written Norwegian, previous or current alcohol or substance abuse, pregnancy, breastfeeding, spinal fracture, malignancy, infection, cauda equina syndrome, rapidly progressive neurologic deficit, or psychiatric, somatic or chronic disorder making the subject unsuitable for inclusion. Subjects recruited from the Østfold Hospital Trust also excluded subjects with prior surgery at the same disc level, lumbar fusion and spinal stenosis. Data from the Department of Neurosurgery at St. Olavs hospital were collected through the Norwegian Registry for Spine Surgery (NORspine), which only included surgical patients.

### Measures

Self-reported questionnaires measured sex (man/woman), age (years), education (≤12 years/> 12 years), height (cm), weight (kg), smoking habits (yes (included occasionally)/no (included former smoker)), daily medication use (yes/no), pain intensity in the past week on a 0–10 numeric rating scale (NRS, 0 = ‘no pain’, 10 = ‘worst pain imaginable’), function affected by pain (Oswestry Disability Index, ODI), pain duration (>3 months/< 3 months) and recovery (pain intensity *< 3 NRS* 12 months after admission/pain intensity ≥ *3 NRS* 12 months after admission). Treatment data (surgical/conservative) was collected by study personnel.

The primary outcome measure for LBP with radiculopathy was back pain intensity 12 months after an acute episode of LBP with radiculopathy. Due to the diversity of clinical symptoms in radiculopathy, two secondary outcome measures were also used; leg pain intensity and ODI 12 months after an acute episode of LBP with radiculopathy. ODI contains ten sections which regard intensity of pain, the influence of pain on the ability to take care of oneself, lift, walk, sit, sleep quality, sexual function, social life and travel. Each section is scored on a scale from 0 to 5 with 0 representing no disability and five representing severe disability. An ODI score was calculated by dividing the summed score by the total possible score which is then multiplied by 100 (0 = no disability and 100 = maximum disability possible).

### Genotyping

Blood samples were obtained at hospital admission in 4 ml EDTA tubes and frozen at −80°C until DNA extraction was performed with QIAamp DNA Blood Kit according to the manufacturer’s protocol (QIAGEN, Valencia, CA, United States). Due to no predesigned TaqMan SNP genotyping assay for *SOX5* rs12310519, it was replaced with a SNP in high LD (rs34616559, *r* ^2^ = 0.95 and D' = 1.00). Genotypes were determined using fast quantitative real time polymerase chain reactions (qPCR) (Gene Amp, PCR System 9,700, Applied Biosystems, California, United States). PCR amplifications were performed using 384-well plates containing 2.25 ml genomic DNA, 2.5 ml TaqPath ProAmp Master Mix (Thermo Fischer scientific Inc., Waltham, MA United States) and 0.25 ml predesigned TaqMan SNP genotyping assay (Thermo Fischer scientific Inc., Waltham, MA United States) and included initialization for 10 min at 95°C, followed by 40 cycles of 15 s at 95°C and 1 min at 60°C. Negative controls containing water were included in every run. Approximately 10% of the samples were re-genotyped with a concordance rate at 100%. Samples with undetermined genotypes were re-genotyped. The overall genotype call rate was 98%.

### Statistical Analysis

Statistical analyses were conducted using SPSS Statistics version 25 (IBM, Armonk, NY). The distribution of the data and residuals was assessed in preliminary analyses by a Shapiro–Wilk test and inspection of descriptive statistics, histograms, boxplots, and Q-Q plots. Deviations from the Hardy-Weinberg equilibrium were assessed with a Chi-square test (*p*-value of <1 × 10^–6^) comparing the observed and expected allele frequencies for each of the SNPs.

Sample size calculations showed that 246 subjects were needed to detect a difference between the three genotypes in each SNP with an effect size of 0.2, a two-sided significance level of 5%, 80% power and an assumption that the risk genotype is present in 5% of the population.

**FIGURE 1:**
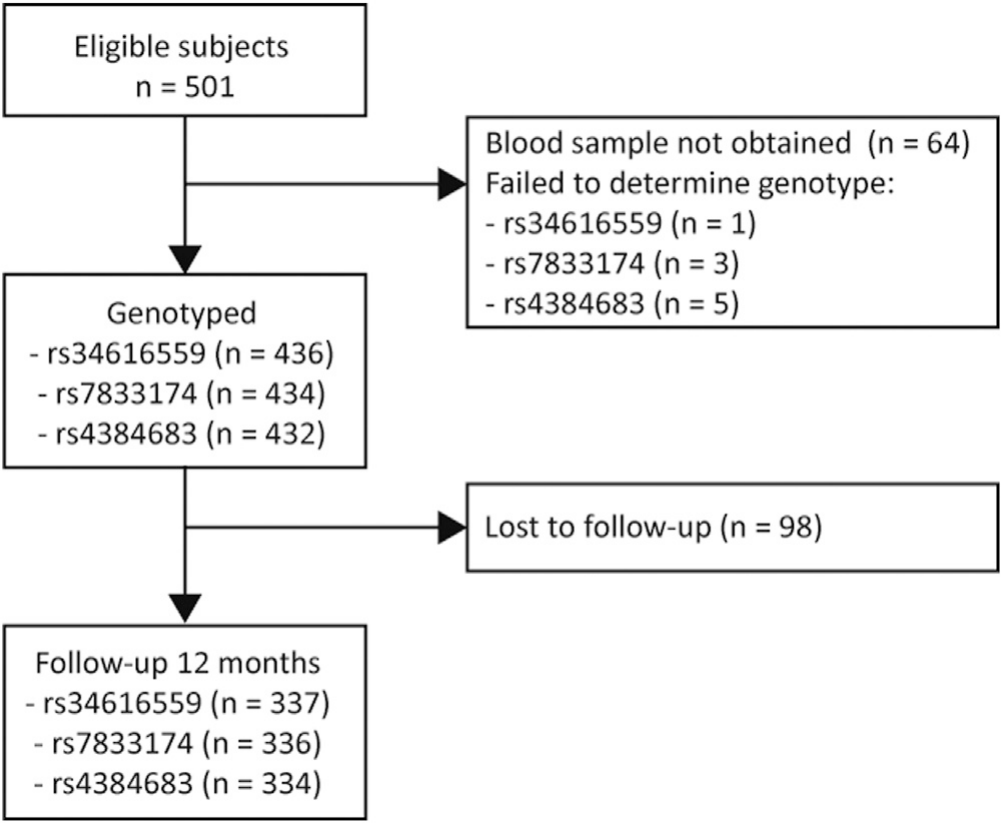
Study flowchart. A total of 334–338 (depending on the different SNPs) was included in the final analysis.

Subjects who responded to the follow-up questionnaire at 12 months were compared with subjects who did not respond with independent sample Student’s t-test for normally-distributed variables, Mann-Whitney *U* test for variables with non-normal distribution, and Chi-square for categorical variables. A Wilcoxon Signed Rank test was used to determine differences between baseline and 12 months for back pain, leg pain and ODI. Missing data were not imputed. The number of subjects with missing genetic data is presented in Figure 1, and the number of missing data for the outcome measures is presented in Table 1.

**TABLE 1:** Sample characteristics for genotyped subjects (*n* = 436) admitted to a secondary health-care institution due to an acute episode of LBP with radiculopathy. Comparison of subjects who responded to the follow-up questionnaire at 12 months with subjects who did not respond.

Characteristics	Total sample (n = 436)	Included subjects (n = 338)	Subjects lost to follow up (n = 98)	*p*-value
Sex, males, n (%)	251 (58.0)	194 (57.2)	57 (60.6)	0.553^a^
Age, years, mean (SD)	42.7 (11.4)	43.8 (11.4)	38.6 (10.7)	<0.001^b^
Education, > 12 years, n (%)	322 (74.5)	255 (75.4)	67 (71.3)	0.412^a^
BMI, kg/m2, mean (SD)	26.0 (4.3)	26.1 (4.3)	25.9 (4.5)	0.788^b^
Smoker, n (%)	76 (17.7)	51 (15.2)	25 (26.6)	0.010^a^
Back pain, 0–10 NRS, median (IQR)	6 (2.0–8.0)	5.5 (2.0–8.0)	6.0 (3.0–8.0)	0.052^c^
Leg pain, 0–10 NRS, median (IQR)	8 (6.0–9.0)	8.0 (6.0–9.0)	8.0 (6.0–9.0)	0.239^c^
ODI, 0–100%, mean (SD)	49.3 (21.6)	49.4 (22.0)	49.1 (20.2)	0.907^b^
Pain duration, back/leg pain >3 months, n (%)	208 (49.1)	156 (47.0)	52 (56.5)	0.106^a^
Daily medication use, n (%)	276 (63.2)	216 (64.5)	60 (63.8)	0.908^a^
Surgical treatment, n (%)	218 (62.1)	160 (62.0)	58 (62.4)	0.952^a^
Back pain 12 months, 0–10 NRS, median (IQR)	1 (0.0–3.0)	1.0 (0.0–3.0)	-	-
Leg pain 12 months, 0–10 NRS, median (IQR)	1 (0.0–3.0)	1.0 (0.0–3.0)	-	-
ODI 12 months, 0–10 NRS, median (IQR)	10 (2.0–22.0)	10.0 (2.0–22.0)	-	-
Recovery, back/leg pain <3 NRS 12 months, n (%)	304 (61.1)	304 (61.1)	-	-
*SOX5* rs34616559, MAF	0.2	0.2	0.1	0.207^a^
*CCDC26/GSDMC* rs7833174, MAF	0.2	0.2	0.2	0.623^a^
*DCC* rs4384683, MAF	0.5	0.5	0.5	0.837^a^

Abberiavations: SD; standard deviation, BMI; body mass index, IQR; interquartile range (25–75%), ODI; oswestry disability index, MAF; minor allele frequency.

a Pearson Chi-Square.

b Independent sample Student’s t-test.

c Mann-Whitney *U* test.

To analyze the association between genotypes and outcome measures, we initially planned to use pre-specified genetic association models (dominant or additive models). However, regression models were considered unsuitable as preliminary analyses revealed that our data violated the assumption of linearity and did also not display non-linear relationships. In addition, our data violated the assumption of normal distributed residuals of the outcome variable, making the non-parametric Kruskal Wallis H test appropriate for investigating associations between each genotype (3 allele groups) of the selected SNPs (*SOX5* rs34616559*, CCDC26/GSDMC* rs7833174 and *DCC* rs4384683) and each outcome measure (back pain, leg pain and ODI). A Bonferroni correction was used to correct for multiple testing of the SNPs (*p* value ≤0.017).

## Results

### Sample Characteristics

Sample characteristics are presented in Table 1. Of the 501 eligible subjects, blood samples were not obtained for 64 subjects due to administrative reasons or because subjects declined to participate. Of the 436 subjects included in the study, 49.1% were admitted to a secondary health care institution due to acute LBP with radiculopathy, and 50.9% were admitted due to an acute worsening of their already persistent LBP with radiculopathy (defined as back and/or leg pain duration >3 months). There was a significant improvement from baseline to 12 months in regards of back pain (*p* < 0.001), leg pain (*p* < 0.001) and ODI (*p* < 0.001). However, 38.9% had back/leg pain intensity ≥3 NRS 12 months after an acute episode of LBP with radiculopathy. In addition to conservative treatment, 62% of the subjects were offered surgical treatment. None of the SNPs deviated significantly from Hardy-Weinberg equilibrium. The minor allele frequency of each SNP also corresponds to frequencies reported in Scandinavian and Western European populations at dbSNP.

The dropout rate at 12 months was 22.5% (Figure 1). There was no significant difference between subjects who responded to the follow-up questionnaire at 12 months and subjects who did not, except for age (43.8 vs. 38.6 years, *p* < 0.001) and smoking habits (15.2% smoker vs. 26.6% smoker, *p* = 0.010) (Table 1).

### Genetic Associations

**FIGURE 2:**
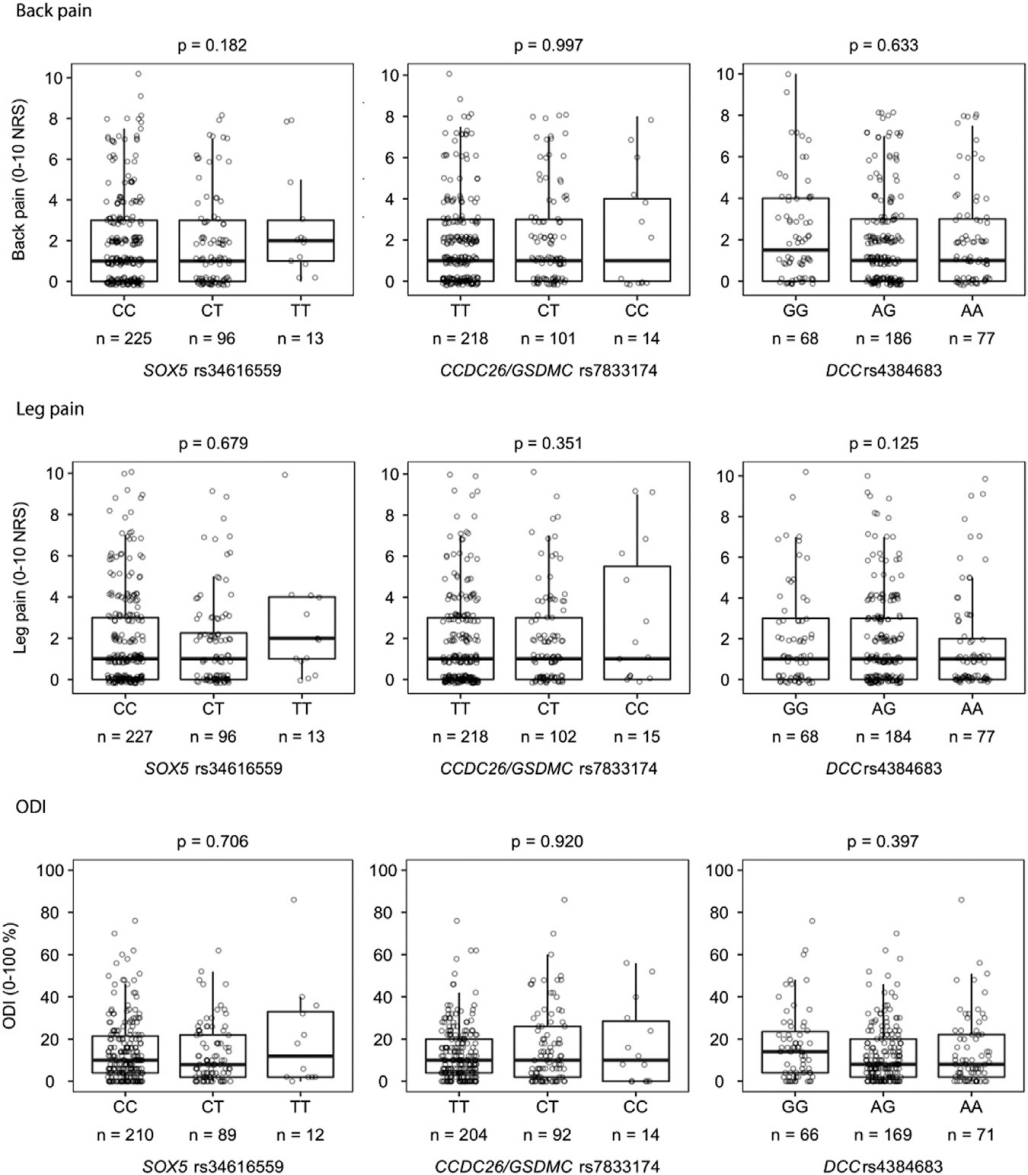
Associations between each genotype (3 allele groups) of the selected SNPs (*SOX5* rs34616559*, CCDC26/GSDMC* rs7833174 and *DCC rs4384683*) and each outcome measure (back pain **(A)**, leg pain **(B)** and ODI **(C)**) analyzed with Kruskal-Wallis H test. Missing data: Back pain (*n* = 3), leg pain (*n* = 1), ODI (*n* = 26). ODI; Oswestry disability index.

There was no statistical significant association between the primary outcome measure, back pain intensity 12 months after an acute episode of radiculopathy, and the selected SNPs (Figure 2). There were also no significant associations between the secondary outcome measures, leg pain intensity and ODI 12 months after an acute episode of radiculopathy, and the selected SNPs (Figure 2).

## Discussion

The recently published GWAS meta-analysis mentioned in the introduction of this paper suggested that the SNPs SOX5 rs34616559, CCDC26/GSDMC rs7833174 and DCC rs4384683 may be linked to chronic back pain (Suri et al., 2018). Since a considerable proportion of the sample used in this GWAS may suffer from radiculopathy, we hypothesized that these SNPs also would have an impact on subjects with LBP with radiculopathy. However, the present study found no associations between LBP with radiculopathy 12 months after an acute episode of LBP with radiculopathy and the selected SNPs. Thus, the present study indicates that the SNPs reported in the GWAS meta-analysis of chronic back pain (*SOX5* rs34616559, *CCDC26/GSDMC* rs7833174 and *DCC* rs4384683) are not of prognostic value in a clinical setting for subjects admitted to a secondary health care institution for an acute episode of LBP with radiculopathy.

*SOX5* is a member of the *SOX* family of transcription factors which are critical for chondrocyte differentiation during embryonic development as well as notochord development (Smits and Lefebvre, 2003; Liu and Lefebvre, 2015; Liu et al., 2017), thus *SOX5* may play an important role in the formation of the spine and the intervertebral discs. *SOX5* has also been associated with cartilage and osteoarthritis in animal studies (Smits et al., 2001; Xu et al., 2012; Budd et al., 2017), but not in human cartilage (Haag et al., 2008). In the GWAS meta-analysis of chronic back pain, chronic back pain was most strongly associated with rs12310519 in the *SOX5* gene (Suri et al., 2018), which may indicate that *SOX5* plays a role in the underlying mechanisms for other chronic back pain conditions than those causing radiculopathy since we did not discover a similar association in the present study. Two GWA studies of LBP with radiculopathy support the present study findings as they do not find any SNPs in the *SOX5* gene associated with LBP with radiculopathy (Lemmelä et al., 2016; Bjornsdottir et al., 2017).

*GSDMC* (Gasdermin C) is a member of the *GSDM* family and contains a conserved two-domain structure (N-terminal and C-terminal domains). When the N-terminal domain is released, it possesses pore-forming activity, which results in loss of cell membrane integrity and release of inflammatory mediators and thereby causes inflammatory cell death (Zheng et al., 2020). *GSDMC* is associated with differential methylation patterns in osteoarthritis-related cartilage and subchondral bone cartilage (Zhang et al., 2016a; Zhang et al., 2016b). *CCDC26* is a long non-coding RNA gene, which modulates retinoic acid, which in turn increases apoptosis (controlled cell death). Absence of apoptosis may result in cancer, while excessive apoptosis may result in a cell death of vital cells causing neurodegenerative diseases (Hirano et al., 2021). The present study did not support the hypothesis that the SNP rs7833174 located between *CCDC26* and *GSDMC* is associated with LBP with radiculopathy 12 months after an acute episode of LBP with radiculopathy. However, *CCDC26*/*GSDMC* rs7833174 have been associated with lumbar microdiscectomy (Bjornsdottir et al., 2017) and lumbar spinal stenosis (Jiang et al., 2020), and a different variant *GSDMC* rs77681114 have been associated with lumbar disc herniation (Wu et al., 2020). Lumbar micro discectomy is a common treatment for lumbar disc herniation (Schoenfeld and Weiner, 2010) and both lumbar disc herniation and lumbar spinal stenosis may cause radiculopathy (Konstantinou and Dunn, 2008). The difference between the present study and the other studies investigating genetic associations for conditions that may cause or be associated with LBP with radiculopathy, is that they use healthy controls as the control group, while the present study only use subjects with LBP with radiculopathy that has recovered. *CCDC26*/*GSDMC* rs7833174 may be associated with LBP with radiculopathy when compared to healthy controls, but may not be able to differentiate between different severities of LBP with radiculopathy.

*DCC* (Deleted in Colorectal *Cancer*) encodes the netrin-1 receptor, which acts as a tumor suppressor when not bound to netrin-1, and as an axon guidance when bound to netrin-1. Netrin-1/*DCC* interactions are involved in pain processing, as it have shown that increased *DCC* expression can cause sprouting of myelinated afferent fibers in the spinal dorsal horn, which may result in mechanical allodynia in animal models (Wu et al., 2016). Netrin-1/*DCC* may also play a role in underlying mechanisms for chronic discogenic back pain, as degenerated intervertebral discs have increased expression of netrin-1 compared to healthy control discs (Bu et al., 2012). However, the present study does not support the hypothesis that this SNP is relevant for LBP with radiculopathy 12 months after an acute episode of LBP with radiculopathy. The two GWA studies of LBP with radiculopathy support the present study findings as they do not find any SNP’s in the *DCC* gene associated with LBP with radiculopathy (Lemmelä et al., 2016; Bjornsdottir et al., 2017).

### Strength and Limitations

While GWA studies based on large samples are best suited for the discovery of novel genetic associations, candidate gene studies may still have a role in the translation of GWAS findings into clinical utility. Due to the need for very large sample sizes in GWAS, the phenotyping and inclusion criteria are often less detailed, leading to a heterogeneous sample. The present study was designed to find SNPs associated with the outcome with an effect size of Cohens *d* 0.2, which is classified as a small to medium effect size (Cohen, 1988). While we did not find an association to LBP with radiculopathy in this study, we cannot rule out true associations with lower effect sizes. We will nevertheless argue that if a common SNP does not have a measurable effect in a carefully phenotyped sample of 300 subjects, it is unlikely that these SNPs will have clinical relevance as prognostic biomarkers.

We did only investigate associations from three SNPs, and cannot exclude that other variants alone or in combination with the selected SNPs could influence the results. Complex phenotypes such as LBP with radiculopathy are typically influenced by a large number of risk variants, each with low effect size (Ioannidis et al., 2006). To achieve meaningful prognostic value it is likely that polygenic risk score based on a high number of variants will be needed. However, high estimates of the relative risk from polygenic risk scores may be difficult to achieve (Wald and Old, 2019; Lewis and Green, 2021).

Back pain was used as the primary outcome measure for LBP with radiculopathy to make the study more comparable with the GWAS and meta-analysis of chronic back pain. To ensure we captured all aspects of LBP with radiculopathy, secondary outcome measures specific for lumbar radiculopathy were also included. In contrast to the GWAS, where chronic back pain was analyzed as a binary trait, we used continuous outcome measures for back pain, leg pain and ODI. We do not believe that this explains the negative results, as using a continuous outcome measure would be expected to increase, rather than decrease, the power of the study. Most of the subjects had low pain intensity scores or low ODI, which could explain the weak or absent association between LBP with radiculopathy and the SNPs. Sensitivity analyses were undertaken, with categorizing outcomes in recovery/non-recovery and in percentage change in outcome scores from baseline to 12 months, but as expected, the sample size in each genotype decreased to a level where we are underpowered to report the findings.

When using a genetic model to predict an outcome, one assumes that the outcome variable is a relatively stable measure. In the present study subjects were asked to rate their pain intensity for the past week, which may fluctuate from week to week, and thereby decrease the precision of the estimates. However, one can assume that such fluctuations are equally distributed across genotypes and thereby should not bias the results.

We have not investigated patient characteristics that may contribute to development of LBP with persistent radiculopathy, other than the selected SNPs. For this subject we refer to a previously published study, where we explore the prognostic value of sociodemographic-, psychosocial- and clinical factors (Fjeld et al., 2017). In this study, we found the following factors significantly associated with leg pain at 12 months; high psychosocial risk according to the Örebro Musculosceletal Pain Questionnaire, not receiving surgical treatment, not actively employed upon admission, and self-reported leg pain recorded 6 weeks after hospital admission (Fjeld et al., 2017). In the present study, subgroup analyses were performed to ensure the results were not influenced by a heterogenous sample in regards to patients receiving surgical treatment and patients reporting high pain intensity at admission (leg/back pain >4 NRS) (Supplementary Table). One may argue that the significant results from our previously published study in combination with the insignificant results from the present study, suggests that sociodemographic-, psychosocial and clinical factors may be more useful as prognostic factors than the selected SNPs alone.

## Conclusion

Our data did not support the hypothesis that LBP with radiculopathy 12 months after an acute episode of LBP with radiculopathy is associated with the selected SNPs; *SOX5* rs34616559*, CCDC26/GSDMC* rs7833174 and *DCC rs4384683*. Therefore, the previously described association between chronic back pain and the selected SNPs appear to be weak or absent in the present cohort. This suggests that the SNPs *SOX5* rs34616559, *CCDC26/GSDMC* rs7833174 and *DCC* rs4384683 are not useful as biomarkers for clinical decision making at an individual level for subjects admitted to a secondary health care institution.

## Data Availability

The original contributions presented in the study are included in the article/Supplementary Material, further inquiries can be directed to the corresponding author.
