# Supplementary material for: Low Back Pain With Persistent Radiculopathy; the Clinical Role of Genetic Variants in the Genes SOX5, CCDC26/GSDMC and DCC
Source: Front Genet. 2022 Jan 24;12:757632. doi: 10.3389/fgene.2021.757632 (PMC8819060; doi:10.3389/fgene.2021.757632)
Supplement: Supplementary file 1 [file Table1.docx]

| Supplementary table. Associations between the selected SNPs and the outcome measures in different subgroups investigated with Kruskal-Wallis H test. Values are presented as median and interquartile range (25 % – 75 %) | | | | | | | | | | | | |
| --- | --- | --- | --- | --- | --- | --- | --- | --- | --- | --- | --- | --- |
|  | *SOX5* rs34616559 | | | | *CCDC26/GSDMC* rs7833174 | | | | *DCC* rs4384683 | | | |
|  | **CC** | **CT** | **TT** | **p-**  **value** | **TT** | **CT** | **CC** | **p-value** | **GG** | **AG** | **AA** | **p-value** |
| Patients receiving surgical treatment (n = 160) | | | | | | | | | | | | |
| Back pain,  0-10 NRS | 1.0  (0.0 – 3.0) | 1  (0.0 – 3.0) | 2.5  (1.3 – 4.5) | 0.343 | 0.0  (0.0 – 5.0) | 1.0  (0.0 – 3.0) | 1.0  (0.0 – 3.0) | 0.702 | 1.0  (0.0 – 2.0) | 1  (0.0 – 3.0) | 1.5  (0.0 – 3.0) | 0.607 |
| Leg pain,  0-10 NRS | 1.0  (0.0 – 3.0) | 1.0  (0.0 – 3.0) | 2.0  (0.5 – 4.0) | 0.330 | 1.0  (0.0 – 7.0) | 1.0  (0.0 – 3.0) | 1.0  (0.0 – 2.0) | 0.503 | 0.0  (0.0 – 1.0) | 1.0  (0.0 – 4.0) | 1.0  (0.0 – 2.3) | 0.500 |
| ODI,  0-100 % | 6.0  (0.0 – 16.0) | 8.0  (2.0 – 23.6) | 18.0  (6.0 – 25.0) | 0.307 | 8.0  (0.0 – 15.0) | 7.0  (0.0 – 20.5) | 7.0  (2.0 – 16.0) | 0.940 | 6.0  (0.0 – 17.0) | 8.0  (2.0 – 20.0) | 14.0  (0.5 – 18.0) | 0.430 |
| Patients reporting high pain intensity at admission (> 4 NRS) (n = 240) | | | | | | | | | | | | |
| Back pain,  0-10 NRS | 1.0  (0.0 – 3.0) | 1  (0.0 – 3.0) | 2.0  (1.0 – 4.0) | 0.229 | 1.0  (0.0 – 4.5) | 1.0  (0.0 – 3.5) | 1.0  (0.0 – 3.0) | 0.982 | 1.0  (0.0 – 3.0) | 1.0  (0.0 – 3.0) | 2.0  (0.0 – 4.0) | 0.716 |
| Leg pain,  0-10 NRS | 0.0  (0.0 – 3.0) | 1.0  (0.0 – 3.0) | 2.0  (0.5 – 4.0) | 0.394 | 1.0  (0.0 – 6.0) | 1.0  (0.0 – 3.6) | 1.0  (0.0 – 3.0) | 0.628 | 1.0  (0.0 – 2.5) | 1.0  (0.0 – 4.0) | 1.0  (0.0 – 3.0) | 0.066 |
| ODI,  0-100 % | 10.0  (0.4 – 22.0) | 8.0  (2.0 – 22.1) | 12.0  (2.0 – 35.0) | 0.702 | 10.0  (0.0 – 32.5) | 10.0  (2.0 – 26.0) | 9.0  (4.0 – 20.0) | 0.784 | 8.0  (2.0 – 22.0) | 10.0  (2.0 – 22.0) | 12.0  (4.0 – 24.0) | 0.443 |
| Abbreviations: ODI; Oswestry disability index | | | | | | | | | | | | |
